# Octa­carbonyldi-μ_2_-hydrido-[μ_3_-(1,3,5-trimethyl­phen­yl)phosphinidene](tri­phenyl­phosphane)-*triangulo*-triruthenium

**DOI:** 10.1107/S1600536812039499

**Published:** 2012-09-22

**Authors:** Taeko Kakizawa

**Affiliations:** a Academic Support Center, Kogakuin University, 1-24-2 Nishi-shinjuku, Shinjuku-ku, Tokyo 163-8677, Japan

## Abstract

In the crystal structure of the title compound, [Ru_3_(C_9_H_11_P)H_2_(C_18_H_15_P)(CO)_8_], the triangular Ru_3_ unit is capped with one mesitylphosphin­idene ligand. In the trigonal–pyramidal Ru_3_P core, one Ru^II^ atom is coordinated by a triphenyl­phosphane ligand in a terminal fashion. Two hydride ligands bridge over two Ru—Ru bonds. These Ru—Ru bonds [2.9400 (4) and 2.9432 (4) Å] are slightly longer than the nonhydride-bridged Ru—Ru bond [2.8146 (4) Å]. The terminal triphenyl­phosphane ligand coordinates to the Ru^II^ atom, which is involved in two hydride bridges.

## Related literature

For related literature, see: Kakizawa *et al.* (2006 ▶); Frediani *et al.* (1997 ▶).
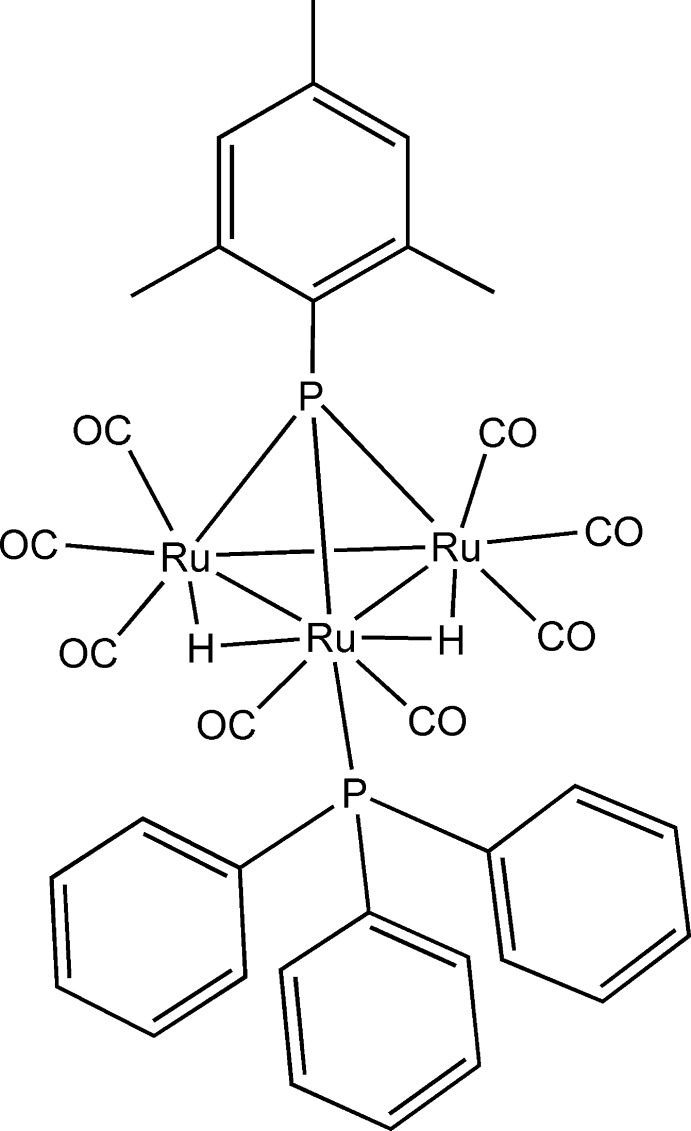


## Experimental

### 

#### Crystal data

[Ru_3_(C_9_H_11_P)H_2_(C_18_H_15_P)(CO)_8_]
*M* *_r_* = 941.72
Triclinic, 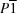

*a* = 11.5954 (5) Å
*b* = 12.0870 (7) Å
*c* = 13.4304 (1) Å
α = 100.224 (2)°
β = 94.6231 (17)°
γ = 95.0167 (13)°
*V* = 1836.44 (13) Å^3^
*Z* = 2
Mo *K*α radiation
μ = 1.35 mm^−1^
*T* = 150 K
0.30 × 0.30 × 0.03 mm

#### Data collection

Rigaku R-AXIS RAPID imaging plate diffractometer
Absorption correction: integration (*NUMABS*; Higashi, 1999 ▶) *T*_min_ = 0.687, *T*_max_ = 0.961
17157 measured reflections
8304 independent reflections
7671 reflections with *I* > 2σ(*I*)
*R*_int_ = 0.035

#### Refinement

*R*[*F* ^2^ > 2σ(*F* ^2^)] = 0.030
*wR*(*F* ^2^) = 0.118
*S* = 1.23
8304 reflections
444 parameters
H atoms treated by a mixture of independent and constrained refinement
Δρ_max_ = 0.74 e Å^−3^
Δρ_min_ = −1.79 e Å^−3^

### 

Data collection: *PROCESS-AUTO* (Rigaku, 1998 ▶); cell refinement: *PROCESS-AUTO*; data reduction: *TEXSAN* (Molecular Structure Corporation & Rigaku, 2000 ▶); program(s) used to solve structure: *SHELXS97* (Sheldrick, 2008 ▶); program(s) used to refine structure: *SHELXL97* (Sheldrick, 2008 ▶); molecular graphics: *ORTEP-3 for Windows* (Farrugia, 1997 ▶); software used to prepare material for publication: *SHELXL97*.

## Supplementary Material

Crystal structure: contains datablock(s) I, global. DOI: 10.1107/S1600536812039499/nc2293sup1.cif

Structure factors: contains datablock(s) I. DOI: 10.1107/S1600536812039499/nc2293Isup2.hkl

Additional supplementary materials:  crystallographic information; 3D view; checkCIF report

## Figures and Tables

**Table 1:** Selected bond lengths (Å)

Ru1—P1	2.3351 (10)
Ru1—P2	2.3896 (9)
Ru2—P1	2.3143 (10)
Ru3—P1	2.3285 (9)

**Fig. 1.**
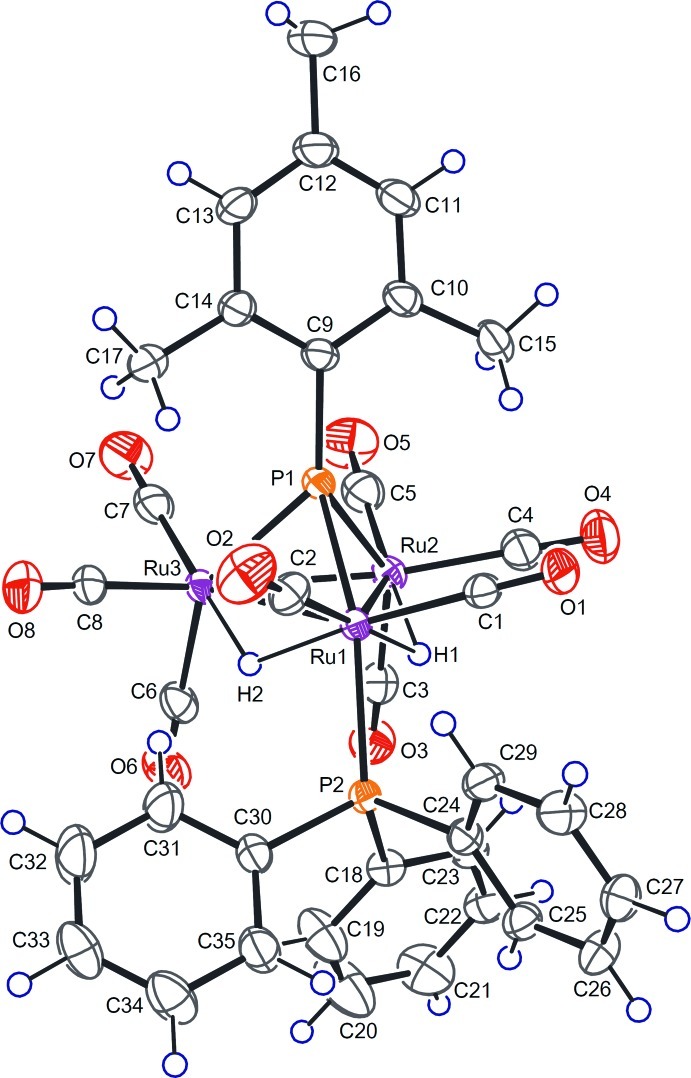
A molecular structure of the title compound, with atom labels and 50% probability displacement ellipsoids for non-H atoms.

## supplementary crystallographic information

### Comment

Previously, we reported the crystal structure of a mesitylphosphinidene-capped triruthenium cluster having a terminal phosphane ligand [Ru_3_(CO)_8_(PH_2_Mes)(µ-H)_2_(µ_3_-PMes)] (Kakizawa *et al.*, 2006). Here we report an additional structure of this type of compound prepared by photo-irradiation of the toluene solution containing [Ru_3_(CO)_9_(µ-H)_2_(µ_3_-PMes)] (Kakizawa *et al.*, 2006) and PPh_3_. The geometry of the title compound is similar to those of the related clusters [Ru_3_(CO)_8_(PH_2_Mes)(µ-H)_2_(µ_3_-PMes)] and [Ru_3_(CO)_8_(PPh_3_)(µ-H)_2_(µ_3_-PPh)] (Frediani *et al.*, 1997) (Fig. 1). In the trigonal pyramidal Ru_3_P core, one Ru atom is co-ordinated by a terminal PPh_3_ ligand. Two hydrido ligands bridge over the Ru(1)–Ru(2) and Ru(1)–Ru(3) bonds. These Ru–Ru bonds (Ru(1)–Ru(2) 2.9400 (4) Å and Ru(1)–Ru(3) 2.9432 (4) Å) are slightly longer than the Ru(2)–Ru(3) bond (2.8146 (4) Å), which has no bridging hydrogen. The existence of two bridging hydrogen atoms was confirmed by the ^1^H NMR spectrum. A signal was observed at -18.32 ppm as a doublet of doublet owing to the coupling with the phosphorus atoms of the µ_3_-PMes (*J*_PH_ = 9.0 Hz) and PPh_3_ (*J*_PH_ = 15.0 Hz) ligands. The ^31^P NMR spectrum shows the signals of µ_3_-PMes and PPh_3_ ligands at a low field (231.3 ppm) and a moderately high field (34.7 ppm), respectively.

### Experimental

All reactions were performed under a dry nitrogen atmosphere or a high vacuum. Toluene and hexane were distilled from sodium-benzophenone ketyl just before use. A toluene solution (2 ml) of [Ru_3_(CO)_9_(µ-H)_2_(µ_3_-PMes)] (25 mg, 0.035 mmol) and PPh_3_ (10 mg, 0.038 mmol) was photolysed for 3 h with a 450 W medium pressure Hg arc lamp with stirring at 6°C. During the photo-irradiation, the evolved CO in the reaction vessel was removed by freeze-pump-thaw cycles every 1 h. After the photolysis, the solvent was filtered and evaporated to dryness under a high vacuum. Recrystallization of the residue from hexane at -30°C gave the title compound (29 mg, 0.031 mmol, 88%) as yellow platelets.

Spectral data for the title compound: ^1^H NMR (300 MHz, CD_2_Cl_2_): δ -18.32 (dd, 2H, *J*_PH_ = 9.0 Hz, *J*_PH_ = 15.0 Hz, µ-H), 2.32 (s, 3H, *p*-CH_3_), 2.77 (s, 6H, *o*-CH_3_), 7.04 (s, 2H, ArH), 7.43–7.47 (m, 15H, P*Ph*). ^31^P NMR (121.5 MHz, CD_2_Cl_2_): δ 34.7 (dt, *J*_PP_ = 116.6 Hz, *J*_PH_ = 15.0 Hz, *P*Ph_3_), 231.3 (dt, *J*_PP_ = 116.6 Hz, *J*_PH_ = 9.0 Hz, *P*Mes). IR νCO (KBr, cm^-1^): 2071 (*s*), 2029 (*vs*), 2012 (*s*), 1988 (*s*), 1968 (*s*), 1965 (*s*). Anal. Calcd for C_35_H_28_O_8_P_2_Ru_3_: C, 44.64; H 3.00. Found: C, 45.02; H, 3.29.

### Refinement

The positions of two hydrogen atoms bridging Ru–Ru bonds were found on the difference Fourier synthesis and refined with isotropic thermal parameters. All other hydrogen atoms were placed at their geometrically calculated positions with C—H = 0.95 and 0.98 Å and with *U*_iso_(H) values of 1.2 and 1.5 times *U*_eq_(C).

### Crystal data

**Table:**

[Ru_3_(C_9_H_11_P)H_2_(C_18_H_15_P)(CO)_8_]	*Z* = 2
*M**_r_* = 941.72	*F*(000) = 928
Triclinic, *P*1	*D*_x_ = 1.703 Mg m^−^^3^
*a* = 11.5954 (5) Å	Mo *K*α radiation, λ = 0.71069 Å
*b* = 12.0870 (7) Å	Cell parameters from 17157 reflections
*c* = 13.4304 (1) Å	θ = 1.6–27.5°
α = 100.224 (2)°	µ = 1.35 mm^−^^1^
β = 94.6231 (17)°	*T* = 150 K
γ = 95.0167 (13)°	Platelet, yellow
*V* = 1836.44 (13) Å^3^	0.30 × 0.30 × 0.03 mm

### Data collection

**Table:**

Rigaku R-AXIS RAPID imaging plate diffractometer	8304 independent reflections
Radiation source: rotation-anode X-ray tube	7671 reflections with *I* > 2σ(*I*)
Graphite monochromator	*R*_int_ = 0.035
ω scans	θ_max_ = 27.5°, θ_min_ = 1.6°
Absorption correction: integration (*NUMABS*; Higashi, 1999)	*h* = −15→15
*T*_min_ = 0.687, *T*_max_ = 0.961	*k* = −15→15
17157 measured reflections	*l* = −17→17

### Refinement

**Table:**

Refinement on *F*^2^	Primary atom site location: structure-invariant direct methods
Least-squares matrix: full	Secondary atom site location: difference Fourier map
*R*[*F*^2^ > 2σ(*F*^2^)] = 0.030	Hydrogen site location: inferred from neighbouring sites
*wR*(*F*^2^) = 0.118	H atoms treated by a mixture of independent and constrained refinement
*S* = 1.23	*w* = 1/[σ^2^(*F*_o_^2^) + (0.0542*P*)^2^ + 4.2474*P*] where *P* = (*F*_o_^2^ + 2*F*_c_^2^)/3
8304 reflections	(Δ/σ)_max_ = 0.001
444 parameters	Δρ_max_ = 0.74 e Å^−^^3^
0 restraints	Δρ_min_ = −1.79 e Å^−^^3^

### Special details

**Table:**

Geometry. All e.s.d.'s (except the e.s.d. in the dihedral angle between two l.s. planes) are estimated using the full covariance matrix. The cell e.s.d.'s are taken into account individually in the estimation of e.s.d.'s in distances, angles and torsion angles; correlations between e.s.d.'s in cell parameters are only used when they are defined by crystal symmetry. An approximate (isotropic) treatment of cell e.s.d.'s is used for estimating e.s.d.'s involving l.s. planes.
Refinement. Refinement of *F*^2^ against ALL reflections. The weighted *R*-factor *wR* and goodness of fit *S* are based on *F*^2^, conventional *R*-factors *R* are based on *F*, with *F* set to zero for negative *F*^2^. The threshold expression of *F*^2^ > σ(*F*^2^) is used only for calculating *R*-factors(gt) *etc*. and is not relevant to the choice of reflections for refinement. *R*-factors based on *F*^2^ are statistically about twice as large as those based on *F*, and *R*- factors based on ALL data will be even larger.

### Fractional atomic coordinates and isotropic or equivalent isotropic displacement parameters (Å^2^)

**Table:**

	*x*	*y*	*z*	*U*_iso_*/*U*_eq_	
Ru1	0.77644 (2)	−0.14550 (2)	0.79228 (2)	0.02048 (9)	
Ru2	0.59156 (3)	−0.33293 (3)	0.77256 (2)	0.02512 (9)	
H1	0.630 (5)	−0.189 (5)	0.822 (4)	0.043 (14)*	
Ru3	0.69300 (3)	−0.29398 (2)	0.59681 (2)	0.02434 (9)	
H2	0.716 (4)	−0.161 (4)	0.664 (4)	0.033 (12)*	
P1	0.78483 (8)	−0.33959 (8)	0.74261 (7)	0.02228 (18)	
P2	0.70618 (8)	0.03502 (8)	0.79537 (7)	0.02088 (18)	
C1	0.8183 (3)	−0.1288 (3)	0.9340 (3)	0.0269 (7)	
C2	0.9265 (4)	−0.0988 (3)	0.7631 (3)	0.0306 (8)	
C3	0.4408 (4)	−0.2860 (4)	0.7289 (3)	0.0320 (8)	
C4	0.5779 (4)	−0.3404 (4)	0.9132 (3)	0.0373 (9)	
C5	0.5459 (4)	−0.4889 (4)	0.7237 (4)	0.0399 (10)	
C6	0.5554 (4)	−0.2355 (4)	0.5397 (3)	0.0333 (9)	
C7	0.6632 (4)	−0.4441 (4)	0.5244 (3)	0.0388 (10)	
C8	0.8053 (4)	−0.2472 (3)	0.5106 (3)	0.0312 (8)	
C9	0.8942 (3)	−0.4344 (3)	0.7652 (3)	0.0252 (7)	
C10	0.8906 (4)	−0.4926 (3)	0.8485 (3)	0.0309 (8)	
C11	0.9668 (4)	−0.5748 (4)	0.8567 (3)	0.0383 (10)	
H3	0.9637	−0.6136	0.9121	0.046*	
C12	1.0461 (5)	−0.6016 (4)	0.7874 (3)	0.0436 (12)	
C13	1.0541 (4)	−0.5387 (4)	0.7105 (3)	0.0365 (10)	
H4	1.1113	−0.5533	0.6645	0.044*	
C14	0.9812 (4)	−0.4547 (3)	0.6983 (3)	0.0287 (8)	
C15	0.8073 (4)	−0.4711 (4)	0.9287 (3)	0.0388 (10)	
H5	0.7298	−0.5082	0.9015	0.058*	
H6	0.8035	−0.3895	0.9485	0.058*	
H7	0.8345	−0.5016	0.9883	0.058*	
C16	1.1215 (7)	−0.6964 (6)	0.7961 (4)	0.070 (2)	
H8	1.1234	−0.7125	0.8652	0.105*	
H9	1.2006	−0.6737	0.7815	0.105*	
H10	1.0891	−0.7643	0.7473	0.105*	
C17	1.0015 (4)	−0.3878 (4)	0.6151 (4)	0.0399 (10)	
H11	1.0771	−0.4012	0.5900	0.060*	
H12	1.0008	−0.3071	0.6422	0.060*	
H13	0.9398	−0.4118	0.5591	0.060*	
C18	0.5479 (3)	0.0334 (3)	0.7800 (3)	0.0245 (7)	
C19	0.4915 (4)	0.0726 (5)	0.7008 (4)	0.0465 (12)	
H14	0.5344	0.0993	0.6509	0.056*	
C20	0.3700 (5)	0.0723 (7)	0.6947 (5)	0.069 (2)	
H15	0.3306	0.0990	0.6403	0.082*	
C21	0.3075 (4)	0.0338 (5)	0.7670 (5)	0.0525 (13)	
H16	0.2255	0.0356	0.7631	0.063*	
C22	0.3638 (4)	−0.0074 (4)	0.8449 (4)	0.0392 (10)	
H17	0.3205	−0.0349	0.8941	0.047*	
C23	0.4834 (4)	−0.0085 (4)	0.8511 (3)	0.0315 (8)	
H18	0.5218	−0.0380	0.9043	0.038*	
C24	0.7488 (3)	0.1366 (3)	0.9136 (3)	0.0232 (7)	
C25	0.6732 (4)	0.2097 (3)	0.9588 (3)	0.0291 (8)	
H19	0.5957	0.2066	0.9288	0.035*	
C26	0.7109 (4)	0.2871 (4)	1.0476 (3)	0.0328 (9)	
H20	0.6588	0.3362	1.0782	0.039*	
C27	0.8236 (4)	0.2928 (4)	1.0915 (3)	0.0347 (9)	
H21	0.8484	0.3448	1.1529	0.042*	
C28	0.9002 (4)	0.2232 (4)	1.0466 (3)	0.0343 (9)	
H22	0.9783	0.2286	1.0759	0.041*	
C29	0.8631 (4)	0.1449 (3)	0.9579 (3)	0.0300 (8)	
H23	0.9160	0.0967	0.9273	0.036*	
C30	0.7549 (3)	0.1124 (3)	0.6979 (3)	0.0267 (7)	
C31	0.7476 (5)	0.2290 (4)	0.7098 (3)	0.0388 (10)	
H24	0.7217	0.2689	0.7700	0.047*	
C32	0.7785 (5)	0.2863 (4)	0.6328 (4)	0.0466 (12)	
H25	0.7731	0.3653	0.6408	0.056*	
C33	0.8165 (5)	0.2301 (5)	0.5459 (4)	0.0471 (12)	
H26	0.8354	0.2698	0.4934	0.056*	
C34	0.8274 (6)	0.1174 (5)	0.5346 (4)	0.0604 (16)	
H27	0.8553	0.0788	0.4748	0.072*	
C35	0.7975 (5)	0.0585 (4)	0.6113 (4)	0.0491 (13)	
H28	0.8067	−0.0198	0.6035	0.059*	
O1	0.8423 (3)	−0.1182 (3)	1.0195 (2)	0.0401 (7)	
O2	1.0180 (3)	−0.0709 (3)	0.7446 (3)	0.0548 (10)	
O3	0.3513 (3)	−0.2646 (3)	0.7010 (3)	0.0446 (8)	
O4	0.5720 (3)	−0.3418 (4)	0.9975 (3)	0.0570 (10)	
O5	0.5191 (4)	−0.5828 (3)	0.6956 (3)	0.0588 (10)	
O6	0.4761 (3)	−0.2000 (3)	0.5072 (3)	0.0483 (9)	
O7	0.6459 (4)	−0.5347 (3)	0.4812 (3)	0.0559 (10)	
O8	0.8706 (3)	−0.2151 (3)	0.4616 (3)	0.0486 (8)	

### Atomic displacement parameters (Å^2^)

**Table:**

	*U*^11^	*U*^22^	*U*^33^	*U*^12^	*U*^13^	*U*^23^
Ru1	0.02143 (15)	0.02131 (15)	0.01840 (14)	0.00346 (11)	0.00047 (10)	0.00297 (10)
Ru2	0.02299 (16)	0.02734 (16)	0.02496 (16)	0.00086 (12)	0.00005 (11)	0.00655 (11)
Ru3	0.02974 (17)	0.02460 (16)	0.01801 (14)	0.00426 (12)	−0.00216 (11)	0.00352 (11)
P1	0.0253 (5)	0.0222 (4)	0.0197 (4)	0.0046 (3)	0.0004 (3)	0.0047 (3)
P2	0.0216 (4)	0.0220 (4)	0.0188 (4)	0.0031 (3)	0.0011 (3)	0.0035 (3)
C1	0.0270 (18)	0.0274 (18)	0.0261 (18)	0.0083 (14)	0.0008 (14)	0.0028 (14)
C2	0.030 (2)	0.0299 (19)	0.0289 (19)	0.0016 (16)	0.0012 (15)	−0.0014 (15)
C3	0.030 (2)	0.035 (2)	0.0291 (19)	−0.0013 (16)	0.0006 (16)	0.0037 (16)
C4	0.030 (2)	0.050 (3)	0.036 (2)	0.0110 (19)	0.0062 (17)	0.0142 (19)
C5	0.038 (2)	0.043 (3)	0.040 (2)	0.0051 (19)	0.0022 (19)	0.0110 (19)
C6	0.040 (2)	0.033 (2)	0.0247 (18)	0.0009 (17)	−0.0045 (16)	0.0056 (16)
C7	0.051 (3)	0.039 (2)	0.027 (2)	0.012 (2)	−0.0044 (18)	0.0065 (17)
C8	0.041 (2)	0.031 (2)	0.0216 (17)	0.0076 (17)	0.0027 (16)	0.0046 (15)
C9	0.0301 (19)	0.0206 (16)	0.0244 (17)	0.0054 (14)	−0.0020 (14)	0.0034 (13)
C10	0.042 (2)	0.0249 (18)	0.0246 (18)	0.0067 (16)	−0.0060 (16)	0.0032 (14)
C11	0.054 (3)	0.032 (2)	0.030 (2)	0.0129 (19)	−0.0071 (19)	0.0073 (17)
C12	0.064 (3)	0.036 (2)	0.031 (2)	0.026 (2)	−0.006 (2)	−0.0003 (17)
C13	0.044 (2)	0.037 (2)	0.0274 (19)	0.0190 (19)	0.0018 (17)	−0.0020 (16)
C14	0.037 (2)	0.0245 (18)	0.0236 (17)	0.0073 (15)	−0.0010 (15)	0.0002 (14)
C15	0.045 (3)	0.048 (3)	0.029 (2)	0.010 (2)	0.0042 (18)	0.0197 (19)
C16	0.113 (6)	0.066 (4)	0.041 (3)	0.064 (4)	0.008 (3)	0.012 (3)
C17	0.048 (3)	0.040 (2)	0.040 (2)	0.021 (2)	0.019 (2)	0.0162 (19)
C18	0.0194 (16)	0.0239 (17)	0.0281 (18)	−0.0018 (13)	−0.0012 (13)	0.0028 (14)
C19	0.025 (2)	0.072 (3)	0.049 (3)	−0.003 (2)	−0.0046 (19)	0.033 (3)
C20	0.029 (3)	0.110 (5)	0.077 (4)	0.002 (3)	−0.012 (3)	0.056 (4)
C21	0.021 (2)	0.066 (3)	0.073 (4)	0.001 (2)	0.001 (2)	0.024 (3)
C22	0.034 (2)	0.034 (2)	0.053 (3)	0.0044 (17)	0.018 (2)	0.0102 (19)
C23	0.029 (2)	0.032 (2)	0.035 (2)	0.0086 (16)	0.0071 (16)	0.0093 (16)
C24	0.0261 (18)	0.0209 (16)	0.0208 (16)	0.0001 (13)	−0.0009 (13)	0.0014 (13)
C25	0.0280 (19)	0.0314 (19)	0.0278 (18)	0.0081 (15)	0.0026 (15)	0.0029 (15)
C26	0.030 (2)	0.035 (2)	0.0297 (19)	0.0040 (16)	0.0047 (16)	−0.0042 (16)
C27	0.043 (2)	0.033 (2)	0.0244 (18)	0.0028 (18)	−0.0016 (16)	−0.0029 (15)
C28	0.032 (2)	0.031 (2)	0.035 (2)	0.0014 (16)	−0.0101 (17)	−0.0008 (16)
C29	0.031 (2)	0.0296 (19)	0.0283 (19)	0.0087 (16)	−0.0010 (15)	0.0005 (15)
C30	0.0261 (18)	0.0313 (19)	0.0236 (17)	0.0028 (15)	0.0017 (14)	0.0083 (14)
C31	0.056 (3)	0.032 (2)	0.028 (2)	0.0008 (19)	0.0010 (19)	0.0073 (16)
C32	0.064 (3)	0.033 (2)	0.041 (3)	−0.011 (2)	−0.006 (2)	0.0131 (19)
C33	0.052 (3)	0.049 (3)	0.043 (3)	−0.010 (2)	0.007 (2)	0.024 (2)
C34	0.092 (5)	0.050 (3)	0.045 (3)	0.003 (3)	0.035 (3)	0.014 (2)
C35	0.075 (4)	0.036 (2)	0.043 (3)	0.008 (2)	0.030 (3)	0.012 (2)
O1	0.0461 (18)	0.0520 (19)	0.0218 (14)	0.0159 (15)	−0.0035 (12)	0.0036 (13)
O2	0.0340 (18)	0.058 (2)	0.072 (3)	−0.0023 (16)	0.0197 (17)	0.0072 (19)
O3	0.0323 (17)	0.058 (2)	0.0420 (18)	0.0115 (15)	−0.0036 (14)	0.0047 (15)
O4	0.051 (2)	0.094 (3)	0.0355 (18)	0.025 (2)	0.0134 (16)	0.0244 (19)
O5	0.067 (3)	0.0280 (18)	0.074 (3)	−0.0083 (17)	−0.001 (2)	0.0006 (17)
O6	0.0413 (19)	0.062 (2)	0.0462 (19)	0.0120 (16)	−0.0067 (15)	0.0231 (17)
O7	0.085 (3)	0.0313 (18)	0.0424 (19)	0.0049 (18)	−0.0144 (19)	−0.0064 (14)
O8	0.059 (2)	0.053 (2)	0.0390 (18)	0.0099 (17)	0.0203 (16)	0.0140 (16)

### Geometric parameters (Å, º)

**Table:**

Ru1—C2	1.875 (4)	C15—H5	0.9800
Ru1—C1	1.897 (4)	C15—H6	0.9800
Ru1—P1	2.3351 (10)	C15—H7	0.9800
Ru1—P2	2.3896 (9)	C16—H8	0.9800
Ru1—Ru2	2.9400 (4)	C16—H9	0.9800
Ru1—Ru3	2.9432 (4)	C16—H10	0.9800
Ru1—H1	1.83 (5)	C17—H11	0.9800
Ru1—H2	1.78 (5)	C17—H12	0.9800
Ru2—C5	1.896 (5)	C17—H13	0.9800
Ru2—C4	1.925 (4)	C18—C19	1.381 (6)
Ru2—C3	1.962 (4)	C18—C23	1.396 (6)
Ru2—P1	2.3143 (10)	C19—C20	1.404 (7)
Ru2—Ru3	2.8146 (4)	C19—H14	0.9500
Ru2—H1	1.75 (5)	C20—C21	1.378 (8)
Ru3—C7	1.889 (5)	C20—H15	0.9500
Ru3—C8	1.925 (4)	C21—C22	1.378 (7)
Ru3—C6	1.956 (4)	C21—H16	0.9500
Ru3—P1	2.3285 (9)	C22—C23	1.384 (6)
Ru3—H2	1.68 (5)	C22—H17	0.9500
P1—C9	1.825 (4)	C23—H18	0.9500
P2—C18	1.828 (4)	C24—C25	1.395 (5)
P2—C24	1.830 (4)	C24—C29	1.396 (5)
P2—C30	1.835 (4)	C25—C26	1.392 (6)
C1—O1	1.141 (5)	C25—H19	0.9500
C2—O2	1.145 (6)	C26—C27	1.380 (6)
C3—O3	1.141 (5)	C26—H20	0.9500
C4—O4	1.143 (6)	C27—C28	1.377 (6)
C5—O5	1.138 (6)	C27—H21	0.9500
C6—O6	1.137 (5)	C28—C29	1.396 (5)
C7—O7	1.136 (6)	C28—H22	0.9500
C8—O8	1.130 (5)	C29—H23	0.9500
C9—C14	1.414 (6)	C30—C35	1.379 (6)
C9—C10	1.423 (5)	C30—C31	1.401 (6)
C10—C11	1.399 (6)	C31—C32	1.395 (6)
C10—C15	1.508 (6)	C31—H24	0.9500
C11—C12	1.378 (7)	C32—C33	1.368 (8)
C11—H3	0.9500	C32—H25	0.9500
C12—C13	1.391 (7)	C33—C34	1.362 (8)
C12—C16	1.516 (6)	C33—H26	0.9500
C13—C14	1.399 (6)	C34—C35	1.400 (7)
C13—H4	0.9500	C34—H27	0.9500
C14—C17	1.513 (6)	C35—H28	0.9500
			
C2—Ru1—C1	94.36 (18)	C14—C9—C10	118.9 (4)
C2—Ru1—P1	97.08 (13)	C14—C9—P1	120.6 (3)
C1—Ru1—P1	100.02 (12)	C10—C9—P1	120.5 (3)
C2—Ru1—P2	94.85 (13)	C11—C10—C9	119.0 (4)
C1—Ru1—P2	97.22 (11)	C11—C10—C15	117.7 (4)
P1—Ru1—P2	158.17 (3)	C9—C10—C15	123.3 (4)
C2—Ru1—Ru2	146.84 (12)	C12—C11—C10	122.6 (4)
C1—Ru1—Ru2	97.77 (13)	C12—C11—H3	118.7
P1—Ru1—Ru2	50.46 (3)	C10—C11—H3	118.7
P2—Ru1—Ru2	113.94 (3)	C11—C12—C13	117.7 (4)
C2—Ru1—Ru3	99.08 (12)	C11—C12—C16	120.4 (5)
C1—Ru1—Ru3	148.99 (12)	C13—C12—C16	121.9 (5)
P1—Ru1—Ru3	50.77 (2)	C12—C13—C14	122.7 (4)
P2—Ru1—Ru3	109.25 (2)	C12—C13—H4	118.7
Ru2—Ru1—Ru3	57.162 (10)	C14—C13—H4	118.7
C2—Ru1—H1	179.1 (18)	C13—C14—C9	118.9 (4)
C1—Ru1—H1	85.1 (17)	C13—C14—C17	118.1 (4)
P1—Ru1—H1	83.7 (17)	C9—C14—C17	123.0 (4)
P2—Ru1—H1	84.6 (17)	C10—C15—H5	109.5
Ru2—Ru1—H1	34.0 (17)	C10—C15—H6	109.5
Ru3—Ru1—H1	81.7 (17)	H5—C15—H6	109.5
C2—Ru1—H2	93.6 (16)	C10—C15—H7	109.5
C1—Ru1—H2	171.7 (16)	H5—C15—H7	109.5
P1—Ru1—H2	81.5 (16)	H6—C15—H7	109.5
P2—Ru1—H2	79.6 (16)	C12—C16—H8	109.5
Ru2—Ru1—H2	76.8 (16)	C12—C16—H9	109.5
Ru3—Ru1—H2	30.9 (16)	H8—C16—H9	109.5
H1—Ru1—H2	87 (2)	C12—C16—H10	109.5
C5—Ru2—C4	95.1 (2)	H8—C16—H10	109.5
C5—Ru2—C3	94.16 (19)	H9—C16—H10	109.5
C4—Ru2—C3	102.67 (18)	C14—C17—H11	109.5
C5—Ru2—P1	95.85 (15)	C14—C17—H12	109.5
C4—Ru2—P1	108.53 (13)	H11—C17—H12	109.5
C3—Ru2—P1	146.14 (13)	C14—C17—H13	109.5
C5—Ru2—Ru3	95.59 (15)	H11—C17—H13	109.5
C4—Ru2—Ru3	159.50 (14)	H12—C17—H13	109.5
C3—Ru2—Ru3	93.97 (13)	C19—C18—C23	119.7 (4)
P1—Ru2—Ru3	52.91 (2)	C19—C18—P2	121.8 (3)
C5—Ru2—Ru1	146.41 (15)	C23—C18—P2	118.5 (3)
C4—Ru2—Ru1	100.92 (15)	C18—C19—C20	119.2 (5)
C3—Ru2—Ru1	110.60 (13)	C18—C19—H14	120.4
P1—Ru2—Ru1	51.09 (2)	C20—C19—H14	120.4
Ru3—Ru2—Ru1	61.476 (10)	C21—C20—C19	120.6 (5)
C5—Ru2—H1	177.5 (18)	C21—C20—H15	119.7
C4—Ru2—H1	82.7 (18)	C19—C20—H15	119.7
C3—Ru2—H1	85.2 (18)	C20—C21—C22	120.0 (4)
P1—Ru2—H1	86.0 (18)	C20—C21—H16	120.0
Ru3—Ru2—H1	86.9 (18)	C22—C21—H16	120.0
Ru1—Ru2—H1	35.7 (18)	C21—C22—C23	119.9 (4)
C7—Ru3—C8	95.2 (2)	C21—C22—H17	120.1
C7—Ru3—C6	97.59 (19)	C23—C22—H17	120.1
C8—Ru3—C6	99.74 (18)	C22—C23—C18	120.6 (4)
C7—Ru3—P1	95.81 (13)	C22—C23—H18	119.7
C8—Ru3—P1	110.90 (12)	C18—C23—H18	119.7
C6—Ru3—P1	145.14 (13)	C25—C24—C29	118.6 (3)
C7—Ru3—Ru2	96.28 (15)	C25—C24—P2	122.7 (3)
C8—Ru3—Ru2	160.67 (12)	C29—C24—P2	118.6 (3)
C6—Ru3—Ru2	94.07 (13)	C26—C25—C24	120.3 (4)
P1—Ru3—Ru2	52.45 (3)	C26—C25—H19	119.8
C7—Ru3—Ru1	146.42 (13)	C24—C25—H19	119.8
C8—Ru3—Ru1	101.19 (12)	C27—C26—C25	120.4 (4)
C6—Ru3—Ru1	108.09 (12)	C27—C26—H20	119.8
P1—Ru3—Ru1	50.97 (2)	C25—C26—H20	119.8
Ru2—Ru3—Ru1	61.362 (10)	C28—C27—C26	120.1 (4)
C7—Ru3—H2	178.1 (17)	C28—C27—H21	119.9
C8—Ru3—H2	86.7 (17)	C26—C27—H21	119.9
C6—Ru3—H2	81.8 (17)	C27—C28—C29	120.0 (4)
P1—Ru3—H2	83.7 (17)	C27—C28—H22	120.0
Ru2—Ru3—H2	82.0 (17)	C29—C28—H22	120.0
Ru1—Ru3—H2	32.9 (17)	C28—C29—C24	120.6 (4)
C9—P1—Ru2	134.38 (14)	C28—C29—H23	119.7
C9—P1—Ru3	133.92 (13)	C24—C29—H23	119.7
Ru2—P1—Ru3	74.63 (3)	C35—C30—C31	118.3 (4)
C9—P1—Ru1	133.56 (13)	C35—C30—P2	121.8 (3)
Ru2—P1—Ru1	78.45 (3)	C31—C30—P2	120.0 (3)
Ru3—P1—Ru1	78.26 (3)	C32—C31—C30	119.8 (4)
C18—P2—C24	103.24 (17)	C32—C31—H24	120.1
C18—P2—C30	103.62 (17)	C30—C31—H24	120.1
C24—P2—C30	102.91 (18)	C33—C32—C31	120.8 (5)
C18—P2—Ru1	115.27 (12)	C33—C32—H25	119.6
C24—P2—Ru1	114.53 (12)	C31—C32—H25	119.6
C30—P2—Ru1	115.62 (13)	C34—C33—C32	120.1 (4)
O1—C1—Ru1	179.2 (4)	C34—C33—H26	120.0
O2—C2—Ru1	179.5 (4)	C32—C33—H26	120.0
O3—C3—Ru2	176.3 (4)	C33—C34—C35	119.9 (5)
O4—C4—Ru2	177.9 (5)	C33—C34—H27	120.0
O5—C5—Ru2	179.1 (5)	C35—C34—H27	120.0
O6—C6—Ru3	179.0 (4)	C30—C35—C34	121.1 (5)
O7—C7—Ru3	179.5 (5)	C30—C35—H28	119.5
O8—C8—Ru3	177.0 (4)	C34—C35—H28	119.5
